# *Vivaria calvasensis*—A new genus and species of Araceae (Araceae: Aroidea: Spathicarpeae) from southern Ecuador

**DOI:** 10.1371/journal.pone.0273867

**Published:** 2022-10-19

**Authors:** Omar Cabrera, Fani Tinitana, Nixon Cumbicus, Paulo Herrera, Aníbal Prina

**Affiliations:** 1 Departamento de Ciencias Biológicas y Agropecuarias, Facultad de Ciencias Exactas y Naturales, Universidad Técnica Particular de Loja,Loja, Ecuador; 2 Facultad de Agronomía, Cátedra de Botánica, Universidad Nacional de La Pampa, Santa Rosa, La Pampa, Argentina; 3 Programa Prometeo, SENESCYT, Azogues, Ecuador; Institute for Biological Research, University of Belgrade, SERBIA

## Abstract

We describe a new genus with a new species belonging to Araceae, from southern Ecuador. *Vivaria calvasensis gen*. *et sp*. *nov*. inhabits semi-arid inter- Andean mountains at altitudes ranging between 1100–1300 m a.s.l. The species belongs to the tribe Spathicarpae, which in Ecuador is represented by two other genera, *Incarum* and *Croatellia*, both typical for humid environments such as montane forests. This new genus is clearly supported by molecular evidence based on the *matK* gene, and morphological traits that separate it from the closely-related genera included in this tribe. The analyzed material was collected during several field campaigns carried out during four years in two populations from Loja province (Calvas and Macará), southern Ecuador, near the border with Peru.

## Introduction

Until 1999, the Araceae family in Ecuador was reported to include 404 species [1], distributed in 21 genera, of which 191 species were endemic [2]. Ulloa and Neill [3] reported 25 additional species and, afterwards [4], added 61 new species, including 7 new records and a taxonomic change, pushing the list in 2011 with a total of 490 species in Ecuador. Recent studies of this highly diverse family added to the Ecuadorian Araceae 28 *Anthurium* species [5–10], 14 *Philodendron* species [11–13], one *Adelonema* [14], two *Caladium*, one *Syngonium* [5] and one *Xanthosoma* species [10]. According to the ’World Checklist of Selected Plant Families’ [15], the Araceae family in Ecuador would be composed of around 747 species.

The Neotropical tribe Spathicarpeae (Araceae, Aroideae) occurs exclusively in South America, being comprised of 13 genera [16, 17]. In Ecuador, this tribe is represented by the genus *Dieffenbachia* [18], *Asterostigma* [19], as well as two newly described species—*Incarum pavonii* (Schott) E. G. Gonç. and *Croatellia integrifolia* (Madison) E. G. Gonç. [16].

Both *Incarum pavonii* and *Croatellia integrifolia* occur in highly-humid montane forests, while *Vivaria calvasensis gen*. *et sp*. *nov*. was collected in dry inter-Andean valleys at medium altitudes, where seasonality is marked and human intervention is very intense, with short cycle crops and extensive grazing. *Vivaria calvasensis gen*. *et sp*. *nov*. grows in semi-arid environments, where individuals spend most of the year in a vegetative state with only one leaf, or dormant, a feature which explains why they often go unnoticed [20].

The use of *matK* for the determination of this new genus and species was based on Goncalvez et al. [16], who reconstructed the phylogeny of the whole tribe based on this gene. It was also more recently used to determine the new monotypic genus *Lorenzia* [21].

## Results

### Phylogenetic analysis

Two DNA sequences from *matK* and two sequences from *trnL-F* were obtained. However, these sequences were respectively identical. Thus, only one sequence of each gene for one sample of the collected plant was used in the phylogenetic analysis together with the 35 sequences per gene obtained from Gonçalves et al. [16].

**Fig 1:**
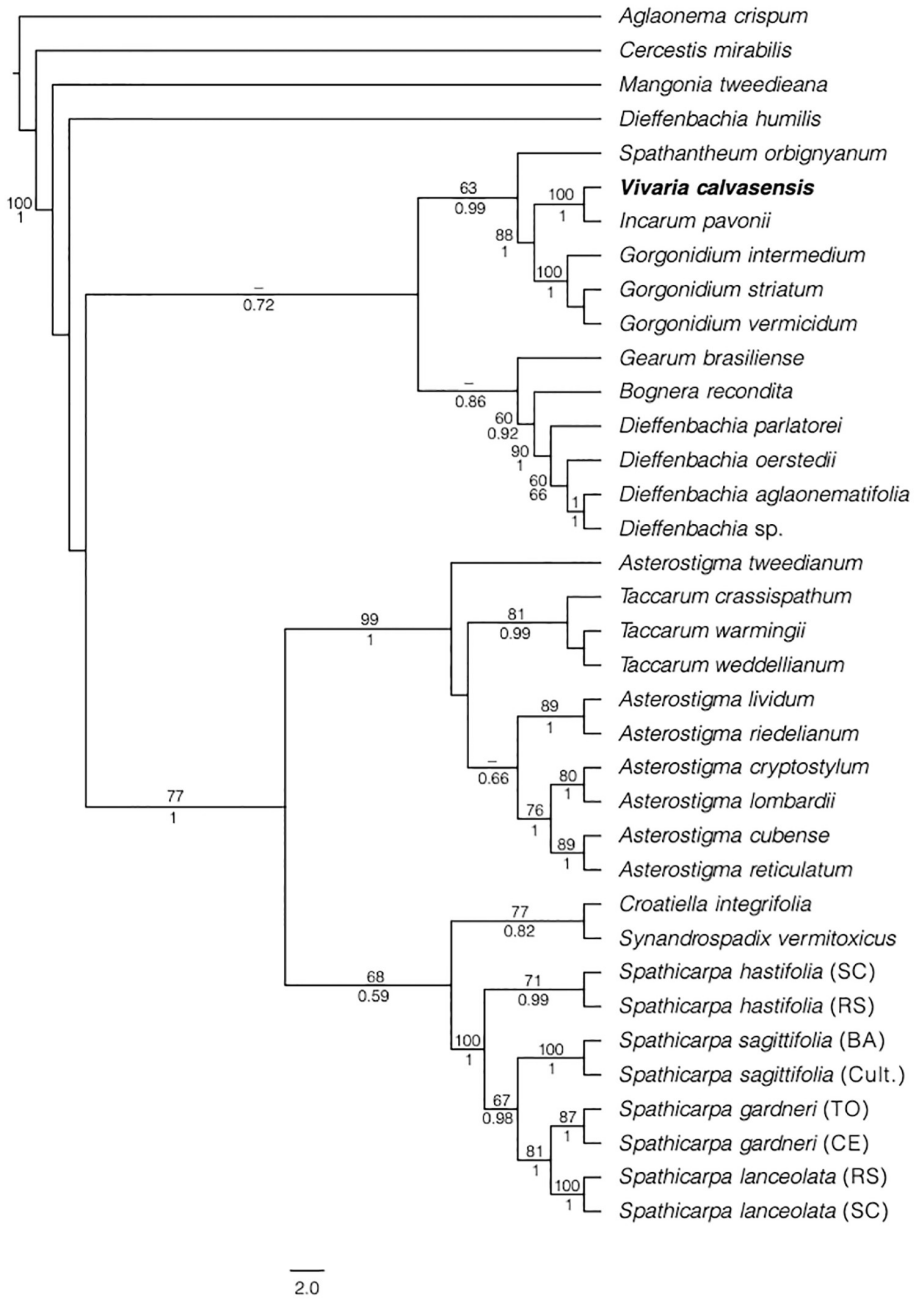
Phylogenetic reconstruction of *Vivaria calvasensis* (tribe Spathicarpeae) for the combined markers *matK* (a) and *trnL-F* (b) using MP and B/MCMC analysis. Values above to the nodes correspond to bootstrap from MP, and values below the nodes correspond to BPP. Only values larger than 50 or 0.5 respectively are shown.

The topology of the concatenated phylogenetic tree (Fig 1) was similar to the one obtained by Gonçalves et al. [16]. The *matK* and *trnL-F* region of our material clustered within the Spathicarpeae tribe in a well-supported clade containing *Gorgonidium* and *Incarum* (Fig 1). Our sequence was located sister to *Incarum*, and this relationship was well supported by MP and BPP analysis (Fig 1). In any case, our sequence was clearly different from *Incarum pavonii* (1.7%) and from *Gorgonidium* (*G*. *intermedium*, 1.6%), which was the genus closest to *Incarum* (Table 1).

**Table 1:** Uncorrected pairwise distances (%), for the combined *matK* and *trnL-F* genes, among the closest *Vivaria calvasensis* genus and species into tribe Spathicarpeae.

	**Species**	**1**	**2**	**3**	**4**	**5**	**6**	**7**	**8**	**9**	**10**	**11**	**12**	**13**	**14**	**15**	**16**	**17**	**18**
1	***Vivaria_calvasensis***																		
2	*Aglaonema_crispum*	3,8																	
3	*Cercestis_mirabilis*	3,4	2,6																
4	*Asterostigma_cryptostylum*	2,1	3,9	3,2															
5	*Asterostigma_cubense*	2,3	3,9	3,3	0,6														
6	*Asterostigma_lividum*	2,3	4,1	3,3	0,9	1,1													
7	*Asterostigma_lombardii*	1,9	3,9	3,2	0,2	0,5	0,9												
8	*Asterostigma_reticulatum*	2,1	3,8	3,1	0,4	0,2	0,8	0,4											
9	*Asterostigma_riedelianum*	2,4	3,9	3,3	0,8	0,9	0,7	0,8	0,7										
10	*Asterostigma_tweedianum*	2,1	3,9	3,2	0,7	0,8	1,1	0,6	0,6	0,9									
11	*Bognera_recondita*	2,0	3,9	3,2	1,8	1,9	1,9	1,8	1,7	1,9	1,9								
12	*Croatiella_integrifolia*	2,6	4,4	3,7	2,3	2,3	2,5	2,2	2,2	2,3	2,1	2,3							
13	*Dieffenbachia_aglaonematifolia*	2,2	4,1	3,2	2,2	2,4	2,3	2,2	2,1	2,3	2,4	1,6	2,3						
14	*Dieffenbachia_humilis*	1,7	3,7	3,0	1,9	2,0	2,0	1,7	1,7	2,0	1,7	1,8	2,3	1,6					
15	*Dieffenbachia_oerstedii*	2,7	4,7	3,9	2,7	2,8	2,7	2,7	2,5	2,7	2,8	2,2	2,9	1,7	2,1				
16	*Dieffenbachia_parlatorei*	2,0	3,9	3,3	2,0	2,1	2,2	2,0	1,9	2,0	2,1	1,4	2,1	1,1	1,5	1,7			
17	*Dieffenbachia_sp*	2,3	4,2	3,3	2,4	2,5	2,4	2,4	2,2	2,4	2,5	1,8	2,4	0,1	1,7	1,8	1,2		
18	*Gearum_brasiliense*	1,8	3,6	2,8	1,6	1,6	1,8	1,5	1,5	1,8	1,7	1,4	2,2	1,7	1,4	2,1	1,4	1,8	
19	*Gorgonidium_intermedium*	1,6	3,8	3,2	2,0	2,1	2,1	1,9	1,9	2,1	1,9	2,1	2,6	2,2	1,8	2,6	1,9	2,3	1,6
20	*Gorgonidium_striatum*	1,6	4,0	3,5	2,3	2,4	2,3	2,1	2,1	2,4	2,2	2,5	2,9	2,6	2,1	3,0	2,3	2,7	2,0
21	*Gorgonidium_vermicidum*	1,5	3,9	3,2	2,1	2,1	2,2	1,8	1,9	2,2	1,9	2,3	2,7	2,4	1,9	2,8	2,1	2,5	1,7
22	*Incarum_pavonii*	1,7	5,0	4,3	3,1	3,2	3,3	2,9	3,1	3,3	3,1	2,9	3,3	3,1	2,9	3,6	2,8	3,2	2,7
23	*Mangonia_tweedieana*	2,1	4,0	3,3	2,3	2,4	2,2	2,3	2,1	2,4	2,2	2,3	2,6	2,1	1,9	2,6	2,0	2,2	1,8
24	*Spathantheum_orbignyanum*	1,2	3,4	2,7	1,4	1,6	1,6	1,3	1,3	1,6	1,4	1,3	1,8	1,4	1,0	2,0	1,2	1,5	1,0
25	*Spathicarpa_gardneri_(TO)*	2,2	4,1	3,4	2,0	2,1	2,4	2,0	1,9	2,1	2,0	2,3	2,3	2,5	2,2	2,8	2,2	2,6	2,2
26	*Spathicarpa_gardneri_(CE)*	2,4	4,2	3,4	2,1	2,2	2,5	2,1	2,0	2,2	2,1	2,4	2,5	2,6	2,4	3,1	2,3	2,7	2,3
27	*Spathicarpa_hastifolia_(SC)*	2,6	4,3	3,8	2,2	2,3	2,6	2,1	2,1	2,3	2,0	2,6	2,4	2,8	2,4	3,1	2,4	2,9	2,4
28	*Spathicarpa_hastifolia_(RS)*	2,6	4,4	3,8	2,2	2,3	2,6	2,2	2,1	2,3	2,1	2,6	2,5	2,8	2,4	3,2	2,5	2,9	2,4
29	*Spathicarpa_lanceolata_(RS)*	2,7	4,4	3,7	2,4	2,6	2,8	2,4	2,3	2,5	2,4	2,8	2,7	2,9	2,7	3,2	2,6	3,0	2,6
30	*Spathicarpa_lanceolata_(SC)*	3,1	4,8	4,0	2,7	2,7	3,1	2,6	2,6	2,8	2,7	3,1	3,0	3,2	3,0	3,7	2,9	3,4	2,8
31	*Spathicarpa_sagittifolia_(BA)*	2,4	4,1	3,4	2,1	2,1	2,4	1,9	1,9	2,3	2,0	2,5	2,5	2,6	2,3	3,0	2,4	2,7	2,1
32	*Spathicarpa_sagittifolia_(Cult*.*)*	2,1	3,8	3,2	1,7	1,9	2,1	1,7	1,6	1,9	1,7	2,2	2,3	2,4	2,0	2,8	2,1	2,5	1,9
33	*Synandrospadix_vermitoxicus*	1,7	3,7	3,1	1,4	1,5	1,6	1,4	1,3	1,6	1,4	1,4	1,6	1,7	1,5	2,2	1,5	1,8	1,3
34	*Taccarum_crassispathum*	1,9	3,7	2,9	0,6	0,7	0,7	0,6	0,4	0,7	0,7	1,6	2,1	1,9	1,6	2,4	1,8	2,0	1,4
35	*Taccarum_warmingii*	2,3	4,2	3,4	1,0	1,1	1,1	0,9	0,9	1,1	1,0	2,1	2,5	2,2	1,9	2,7	2,2	2,4	1,8
36	*Taccarum_weddellianum*	2,1	3,9	3,2	0,8	0,9	1,0	0,8	0,7	1,0	0,8	1,9	2,1	2,0	1,8	2,5	1,9	2,2	1,7
	**Species**	**19**	**20**	**21**	**22**	**23**	**24**	**25**	**26**	**27**	**28**	**29**	**30**	**31**	**32**	**33**	**34**	**35**	
1	***Vivaria_calvasensis***																		
2	*Aglaonema_crispum*																		
3	*Cercestis_mirabilis*																		
4	*Asterostigma_cryptostylum*																		
5	*Asterostigma_cubense*																		
6	*Asterostigma_lividum*																		
7	*Asterostigma_lombardii*																		
8	*Asterostigma_reticulatum*																		
9	*Asterostigma_riedelianum*																		
10	*Asterostigma_tweedianum*																		
11	*Bognera_recondita*																		
12	*Croatiella_integrifolia*																		
13	*Dieffenbachia_aglaonematifolia*																		
14	*Dieffenbachia_humilis*																		
15	*Dieffenbachia_oerstedii*																		
16	*Dieffenbachia_parlatorei*																		
17	*Dieffenbachia_sp*																		
18	*Gearum_brasiliense*																		
19	*Gorgonidium_intermedium*																		
20	*Gorgonidium_striatum*	0,6																	
21	*Gorgonidium_vermicidum*	0,3	0,5																
22	*Incarum_pavonii*	2,4	2,7	2,4															
23	*Mangonia_tweedieana*	2,1	2,3	2,1	3,0														
24	*Spathantheum_orbignyanum*	1,1	1,4	1,3	2,1	1,6													
25	*Spathicarpa_gardneri_(TO)*	2,5	2,6	2,6	3,5	2,6	1,9												
26	*Spathicarpa_gardneri_(CE)*	2,6	2,9	2,7	3,6	2,7	2,1	0,3											
27	*Spathicarpa_hastifolia_(SC)*	2,6	2,8	2,6	3,7	2,5	2,0	1,8	1,8										
28	*Spathicarpa_hastifolia_(RS)*	2,7	2,9	2,8	3,6	2,7	2,1	1,5	1,6	1,3									
29	*Spathicarpa_lanceolata_(RS)*	2,9	3,1	2,9	3,6	3,0	2,4	0,9	0,9	2,1	1,2								
30	*Spathicarpa_lanceolata_(SC)*	3,2	3,5	3,0	3,8	3,3	2,7	1,3	1,2	2,4	1,6	0,4							
31	*Spathicarpa_sagittifolia_(BA)*	2,6	2,7	2,5	3,5	2,6	2,0	1,0	1,2	1,8	1,4	1,4	1,7						
32	*Spathicarpa_sagittifolia_(Cult*.*)*	2,3	2,4	2,3	3,3	2,3	1,7	0,8	0,9	1,4	1,1	1,2	1,6	0,3					
33	*Synandrospadix_vermitoxicus*	1,8	2,2	2,0	2,5	1,9	1,1	1,6	1,8	2,0	1,9	2,1	2,4	1,8	1,5				
34	*Taccarum_crassispathum*	1,9	2,1	1,9	3,1	1,9	1,3	1,8	1,9	2,0	2,0	2,2	2,5	1,9	1,6	1,2			
35	*Taccarum_warmingii*	2,2	2,4	2,3	3,3	2,3	1,6	2,3	2,4	2,4	2,5	2,7	3,1	2,4	2,1	1,7	0,6		
36	*Taccarum_weddellianum*	2,1	2,4	2,2	3,2	2,1	1,6	1,9	2,1	2,0	1,9	2,4	2,7	2,0	1,7	1,4	0,4	0,8	

### Taxonomic treatment

Liliopsida Scopoli, 1760

Alismatales Dumortier, 2009

Araceae Juss, 1789

Aroideae Arnott, 1832

*Vivaria* O. Cabrera, Tinitana, Cumbicus, Prina & Herrera, 2021

**Fig 2:**
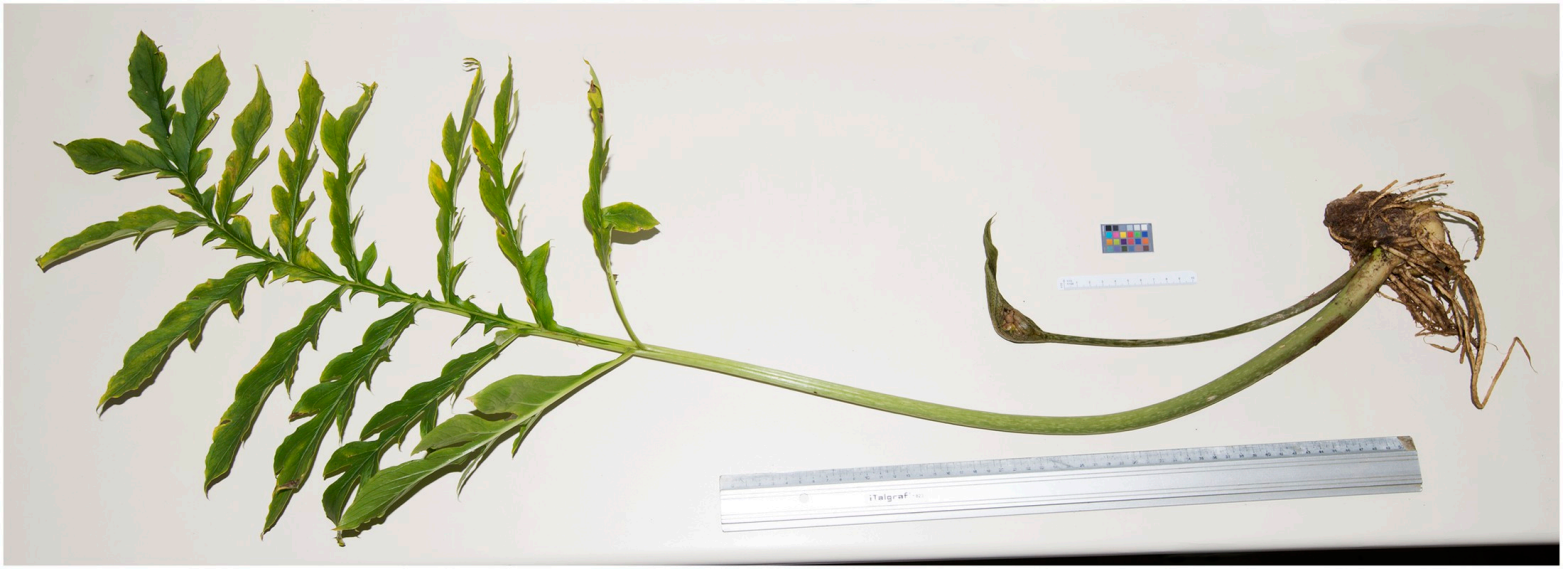
*Vivaria calvasensis* adult individual in which a mature leaf, infructescence and bulb can be observed.

**Fig 3:**
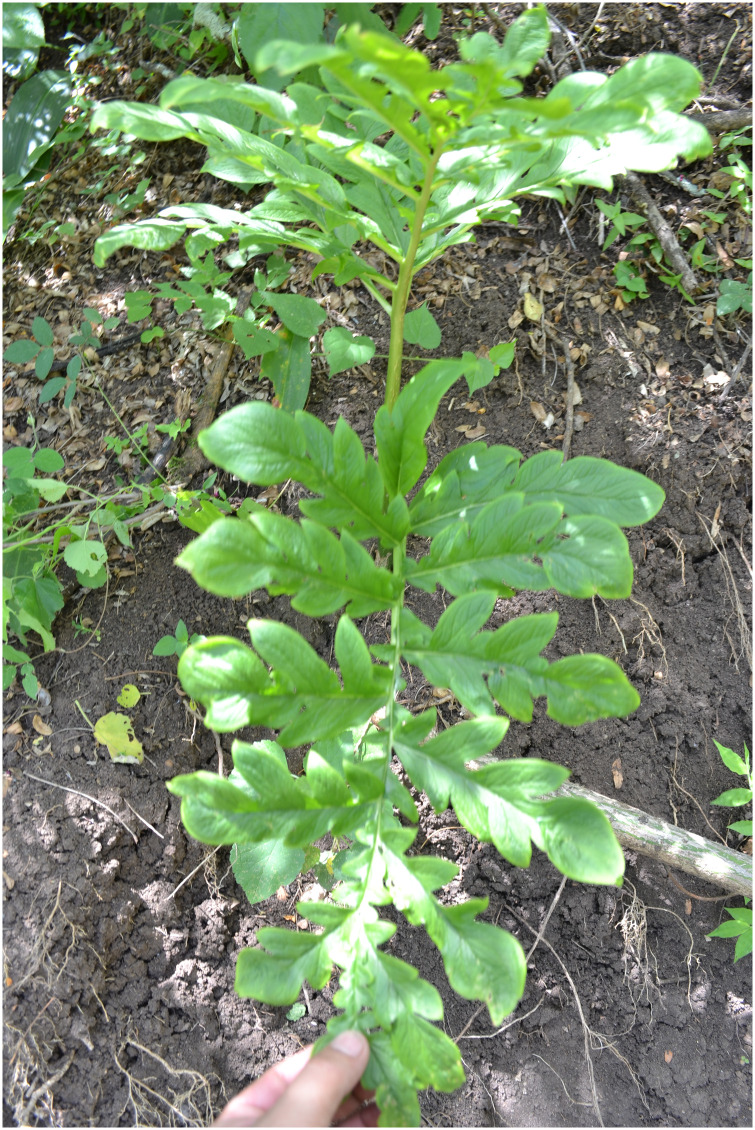
Mature leaf of *Vivaria calvasensis* collected in its natural habitat.

**Fig 4:**
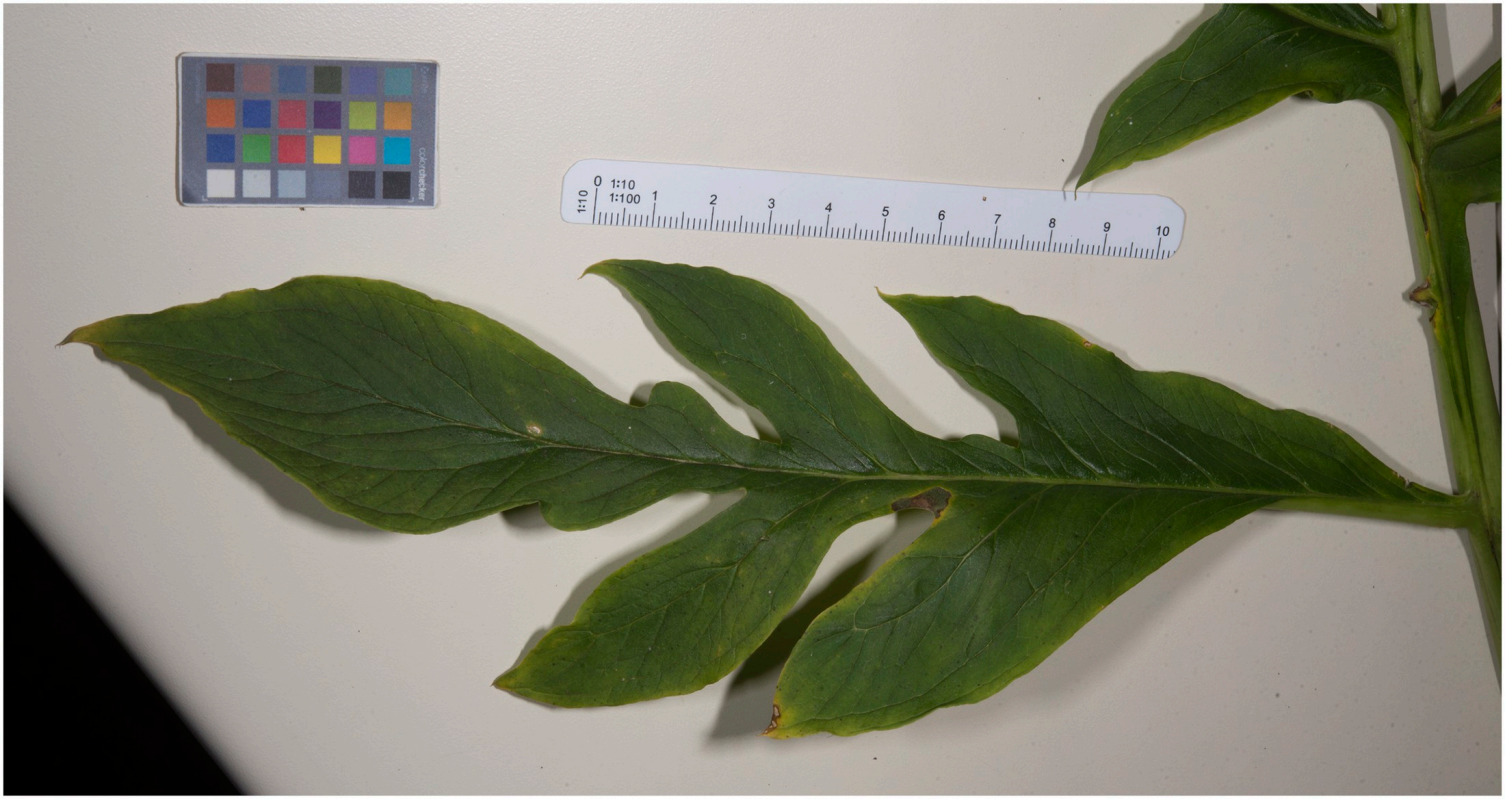
Pinna taken from the central section of a mature *Vivaria calvasensis* leaf.

**Fig 5:**
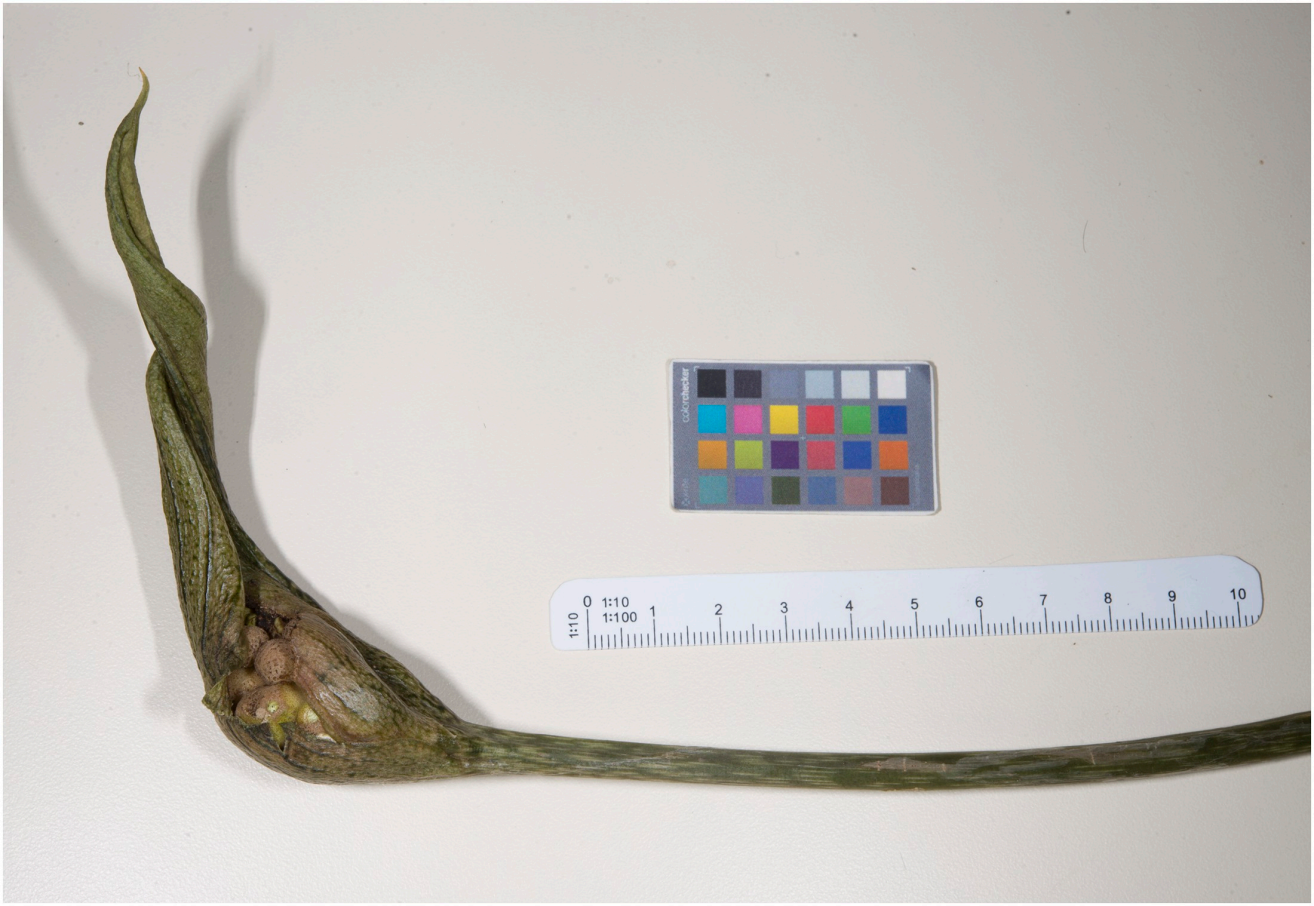
Upper section of the inflorescence of *Vivaria calvasensis*.

**Fig 6:**
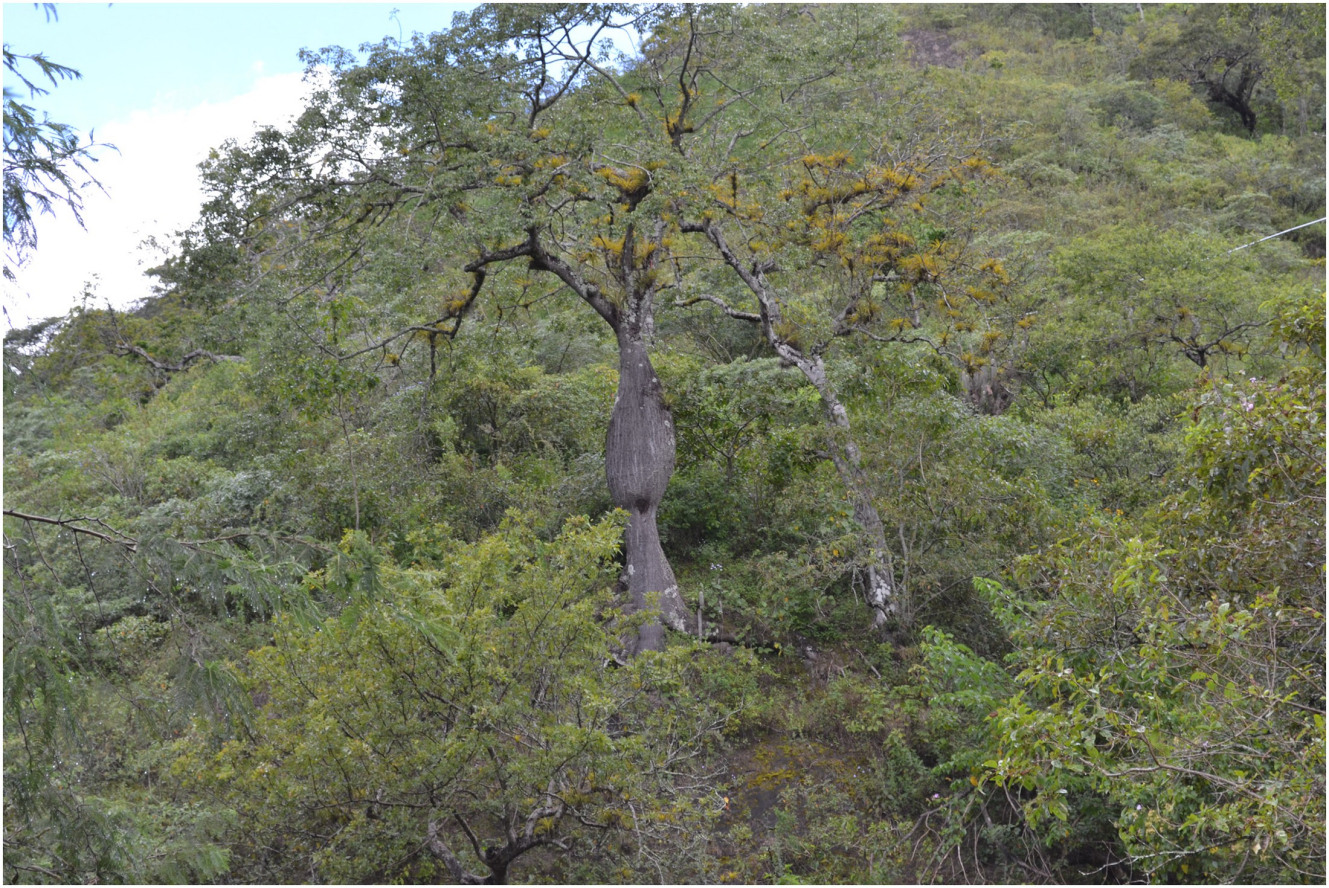
Panoramic view of the dry scrub, habitat of *Vivaria calvasensis* at the end of the rainy season.

***Vivaria calvasensis*** O. Cabrera, Tinitana, Cumbicus, Prina & Herrera ***gen*. *et sp*. *nov***. (Figs 2–6).

*Vivaria O*. Cabrera, Tinitana, Cumbicus, Prina & Herrera, gen. nov.: 77304415–1 *Vivaria calvasensis* O. Cabrera, Tinitana, Cumbicus, Prina & Herrera sp. nov.: 77304416–1.

#### Etymology

The generic name is dedicated in honor of Ing. Francisco Vivar who is a recognized professor of Botany, LOJA Herbarium and “Reinaldo Espinosa” Botanical Garden founder. The specific epithet refers to the place of collection (Canton Calvas, Loja province, south west of Ecuador).

### Holotype

Type. ECUADOR, Loja province, Calvas canton, 21 km (by road) to “Bella María” locality 04°11´59” S, 79°36´56” W (datum WGS84), 1200 m a.s.l., 30.01.2013. Omar Cabrera, Fani Tinitana and Nixon Cumbicus 830, HUTPL.

#### Paratypes (2 specimens)

ECUADOR, Loja province, Calvas canton, 19 km (by road) to “Bella María” locality, 04°13´20” S, 79°36´22” W (datum WGS84), 1222 m a.s.l., 24.03.2016. Nixon Cumbicus, Omar Cabrera, Fani Tinitana, 1654, HUTPL

ECUADOR, Loja province, Calvas canton, 19 km (by road) to “Bella María” locality, 04°13´20” S, 79°36´22” W (datum WGS84), 1222 m a.s.l., 24.03.2016. Nixon Cumbicus, Omar Cabrera, Fani Tinitana, 1655, HUTPL

**Fig 7:**
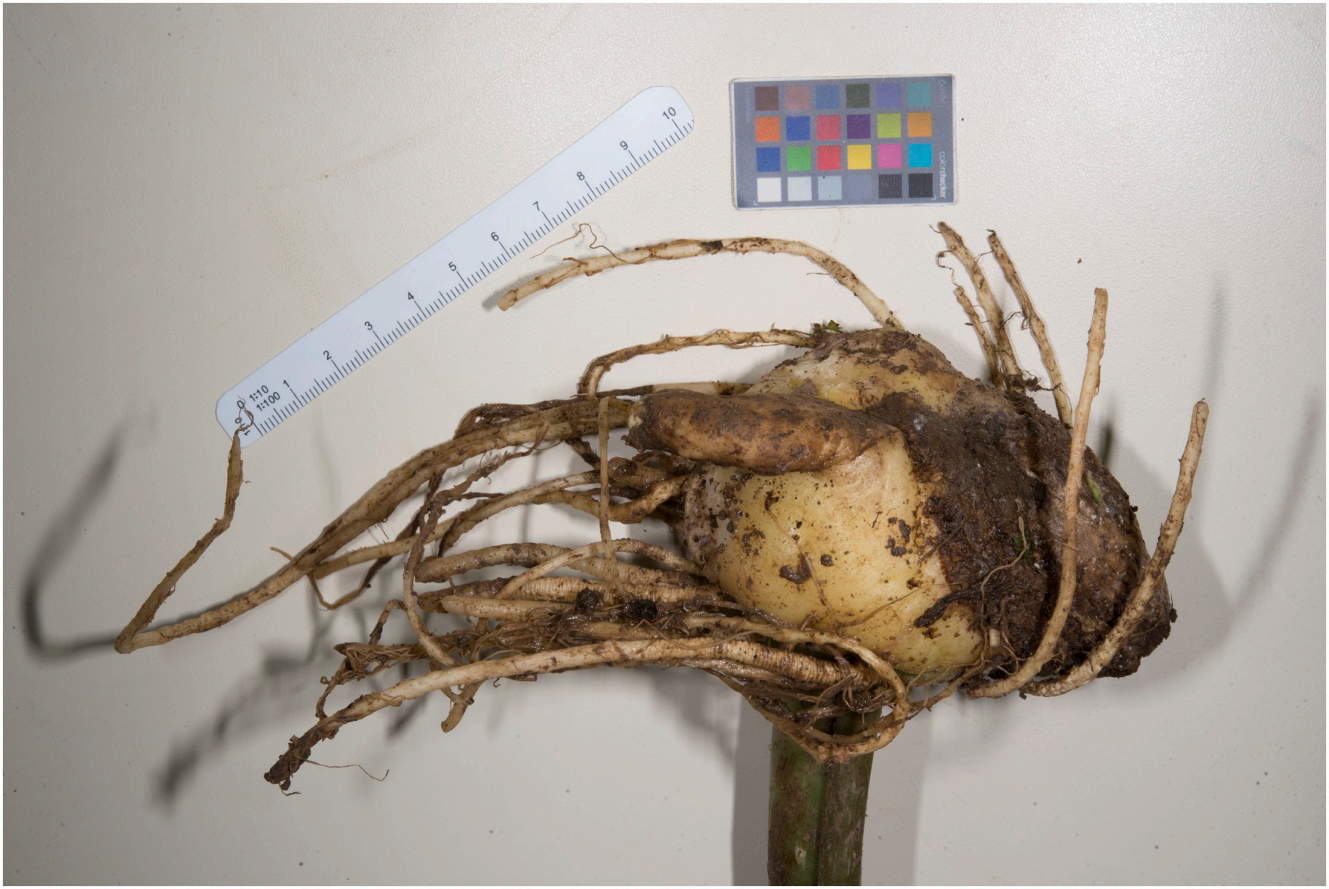
Rhizome and roots of an adult individual collected.

**Fig 8:**
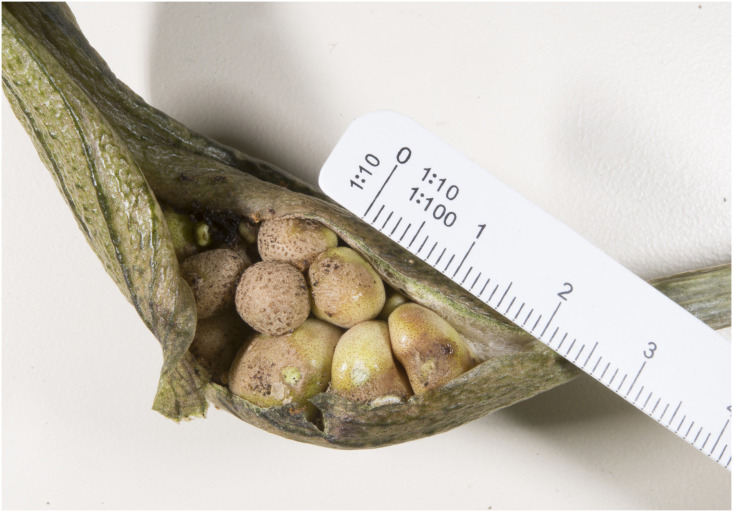
Unfruitfulness and unripe berries collected.

**Fig 9:**
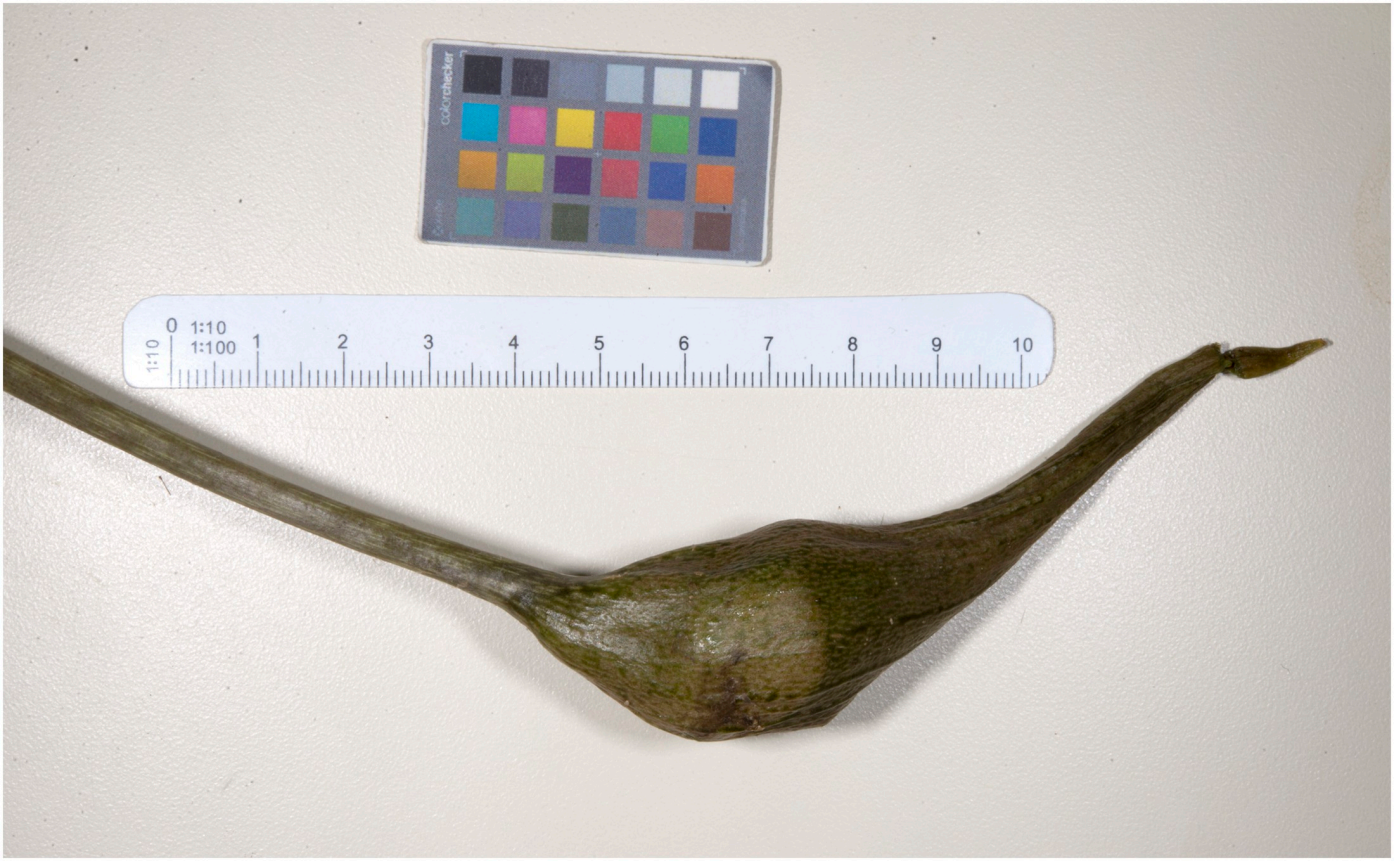
Floral tube and closed immature infructescence.

**Fig 10:**
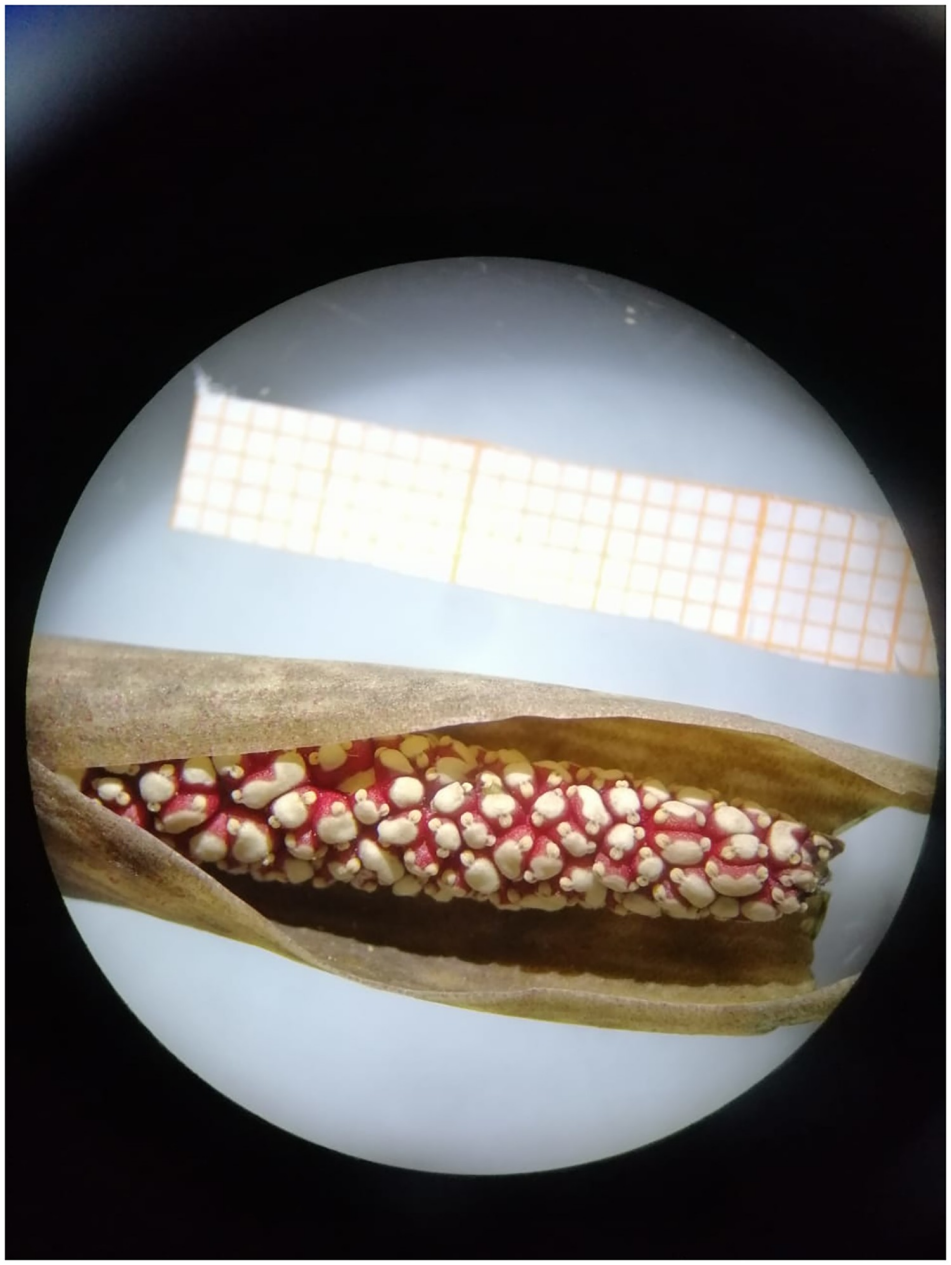
Spathe and spadix.

Figs 7–10. **Holotype of *Vivaria calvasensis gen et*. *sp*. *nov*. (HUTPL)**

### Diagnosis

**Fig 11:**
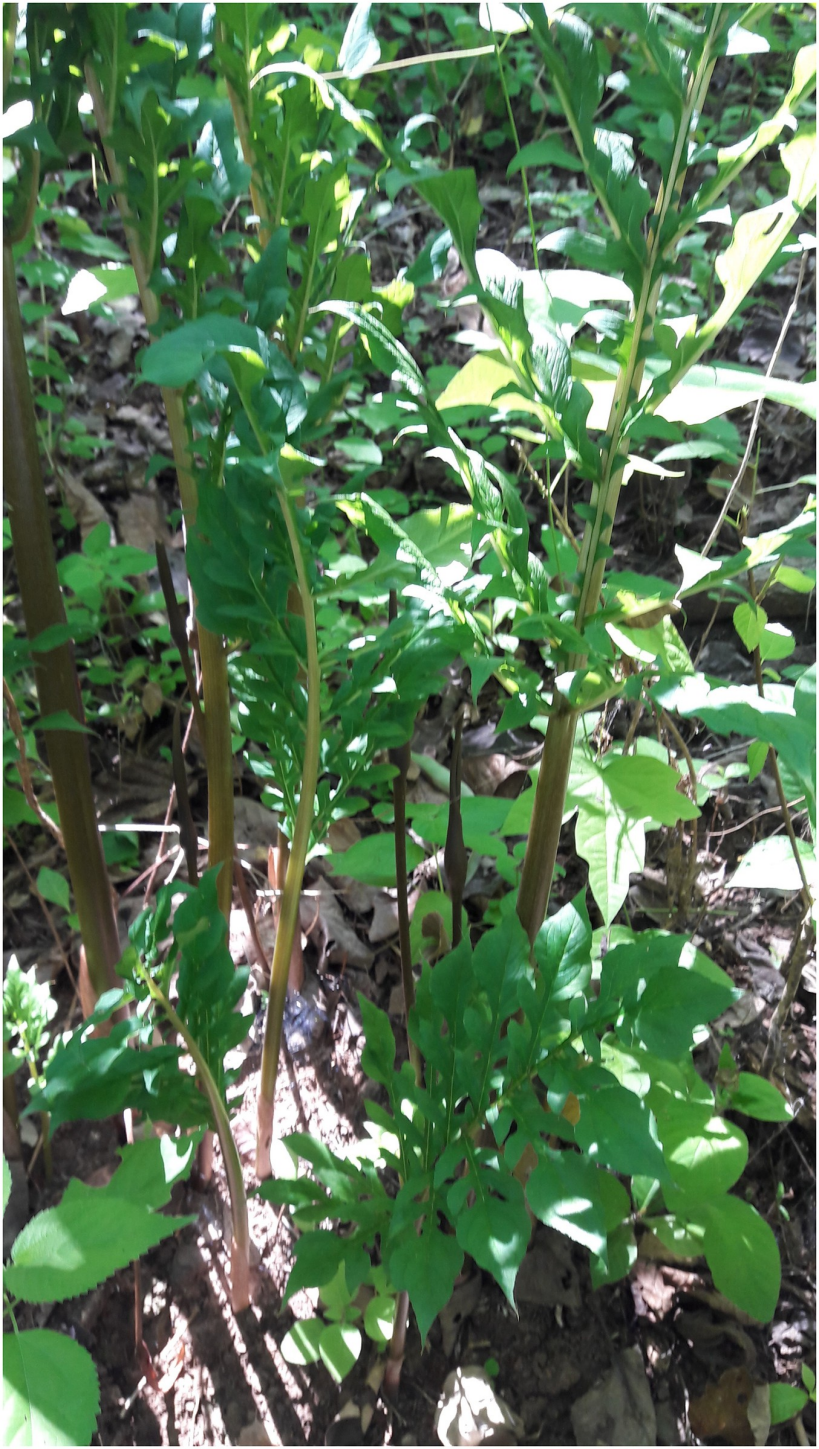
Seedlings and medium plants of *Vivaria calvasensis*, note the shape of the leaves.

**Fig 12:**
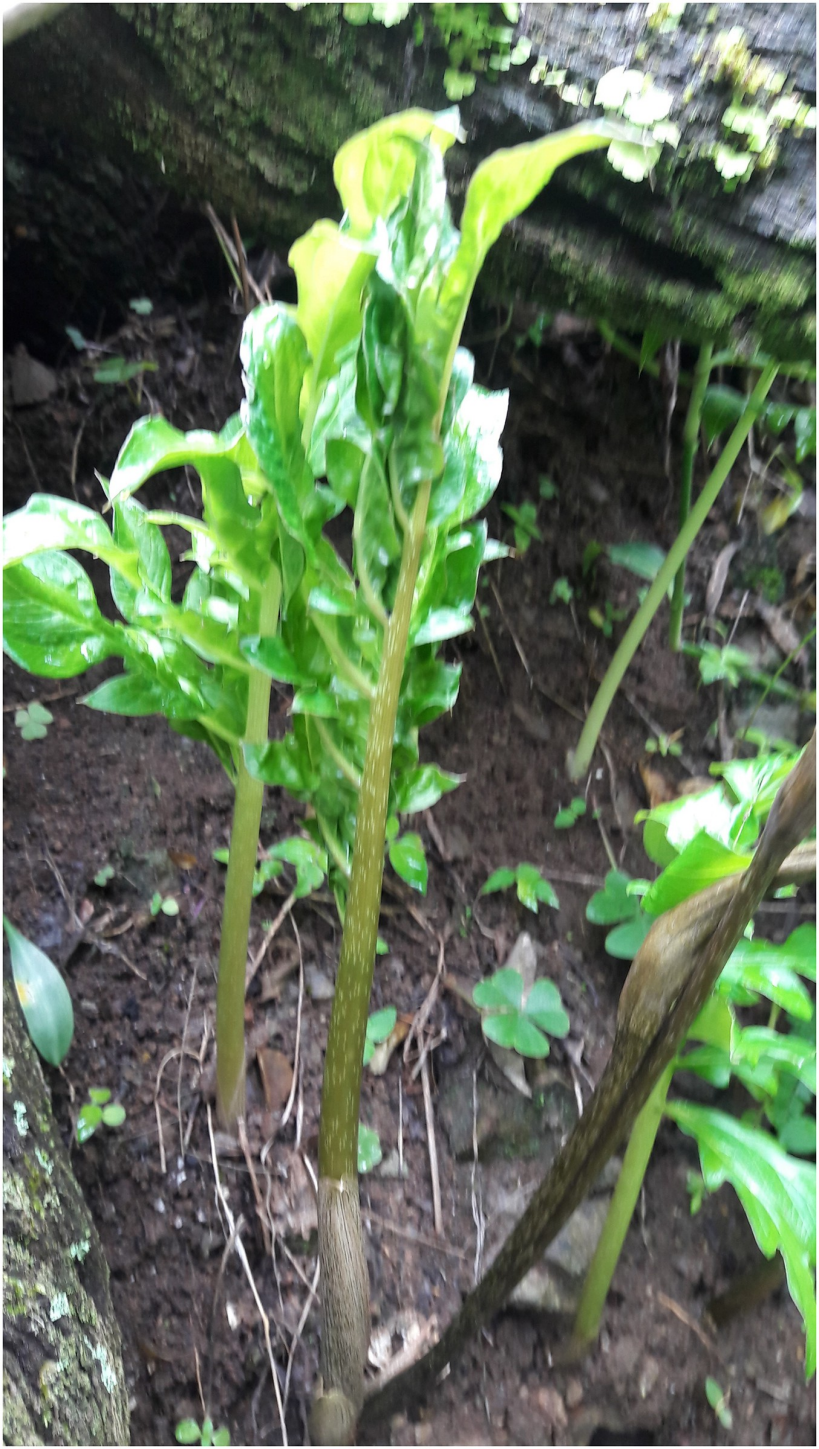
Seedlings of *Vivaria calvasensis*, note the shape of the leaves, kept until they reach maturity.

Geophyte, approximately 1.3–1.5 m high, 1–2 leaves growing during the rainy season. Rounded tuber flattened at base and apex, 16–24 cm in circumference, thickness of 3.5 to 5 cm. Leaf petiole 39–63 cm long, 1.4–1.7 cm in diameter at the base and 1 cm at the apex, leaf-blade pinnatipartite, 44–49 cm long × 56–62.5 cm wide, green; pinnae sub opposed, 7 lateral lobes 2–6 cm long, the pinnatipartite primaries one on each side and one terminal, smooth surface with elongated lines 1 mm wide (Figs 11 and 12). Laminar cataphyll light brown and deciduous with the maturity of the leaf. Solitary inflorescence; peduncle 25–29 cm long and 0.5–0.8 cm in diameter. Spathe cymbiform, herbaceous, brown when immature and green at maturity, with small longitudinal stripes of light brown colour, 10–16 × 3.5 cm in diameter.

**Fig 13:**
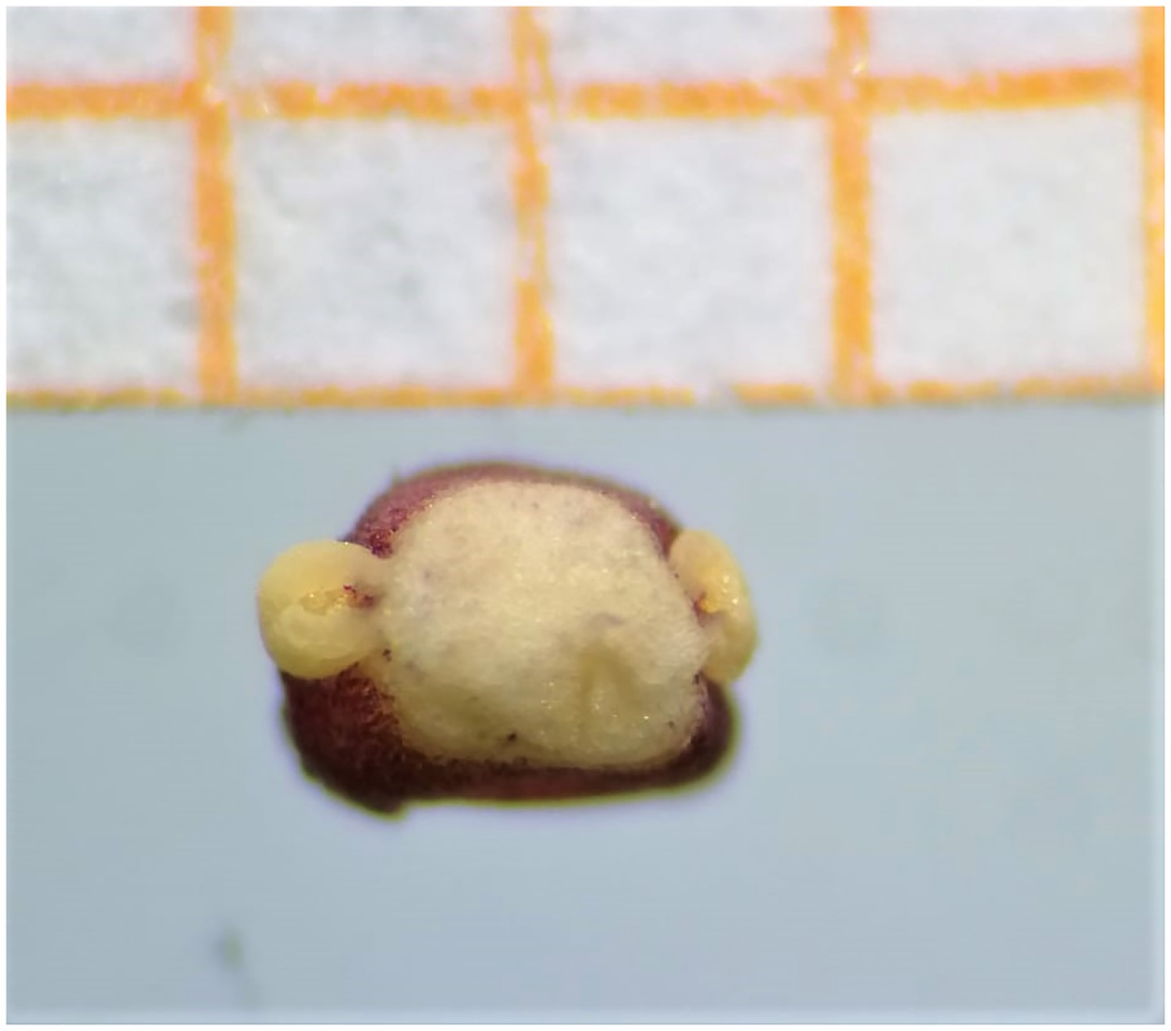
Synandrium of *Vivaria calvasensis*.

Spadix sessile of 3.0–3.5cm long; female zone of 2.0–2.5 × 0.2–0.5cm in diam, obliquely adnate to the spathe, until the maturity of the fruits; male zone of 0.5–1.5cm long. Flowers pistillate with the ovary widely elliptical, 1–1.2 × 1.2–1.5 mm, with three locules, each locule with a single ovule. Staminated flowers seen (Fig 13). Infructescence a pseudobaya, white-greenish 1.0–1.5 cm.

**Typus**: *Vivaria calvasensis*. O. Cabrera, F. Tinitana, N. Cumbicus, A. Prina & P. Herrera.

### Comparisons with similar species

*Vivaria* is a monotypic genus, closely related to *Incarum* and *Gorgonidium*. We refer to 35 of 46 characters previously used by Goncalves [16] to build the taxonomy of the tribe Spathicarpeae which we adopted to taxonomically place *Vivaria* within this tribe. Of the 35 characters used, 22 are morphological; in character 8 "basic leaf pattern", we include a new description adding the state "pinnatipartite", which is exclusive to *Vivaria* gen nov. assigning the number 3 in the corresponding character. In character 34 we also include a new category of the "seedling leaf" state by adding the "pinnate" state and assigning it the number 2. The other 13 characters used correspond to characteristics of male flowers that reinforce the separation of *Vivaria* gen nov. of the most closely related genera in the clade.

A comparison of phenotypic traits between *V*. *calvasensis* and the four most similar species is given in Table 2.

**Table 2:** Comparison matrix of phenotypic characters between *Vivaria calvasensis* and the most closely related species based on the most important morphological characters within the tribe (Table 3).

	Characters (*)																	
**Species**	**1**	**2**	**3**	**4**	**5**	**6**	**7**	**8**	**9**	**10**	**11**	**12**	**13**	**14**	**15**	**16**	**17**	**18**
*Gorgonidium intermedium*	1	1	2	0	1	0	0	0	0	0	2	0	0	1	0	0	0	0
*Gorgonidium striatum*	1	1	2	0	1	0	0	0	1	0	0	0	0	1	0	3	0	0
*Gorgonidium vermidicum*	1	1	2	0	1	0	0	0	1	0	1	0	0	1	0	0	0	0
*Incarum pavoni*i	1	1	2	0	1	0	0	0	0	1	1	0	0	1	0	1	1	0
***Vivaria calvasensis***	**1**	**1**	**2**	**0**	**1**	**0**	**0**	**3**	**1**	**1**	**2**	**1**	**0**	**1**	**1**	**3**	**0**	**0**
	Characters (*)																	
**Species**	**19**	**20**	**21**	**22**	**23**	**24**	**25**	**26**	**27**	**28**	**29**	**30**	**31**	**32**	**33**	**34**	**35**	
*Gorgonidium intermedium*	0	0	1	2	1	1	0	0	2	0	?	?	?	?	0	0	1	
*Gorgonidium striatum*	0	0	1	2	1	2	0	1	2	0	?	?	?	?	0	0	1	
*Gorgonidium vermidicum*	0	0	1	1	1	1	0	0	2	0	2	2	1	1	0	0	1	
*Incarum pavoni*i	0	0	1	1	1	1	0	0	2	0	2	2	0	1	0	0	1	
***Vivaria calvasensis***	**1**	**0**	**0**	**0**	**0**	**1**	**0**	**0**	**2**	**0**	**2**	**1**	**0**	**1**	**0**	**2**	**1**	

**Fig 14:**
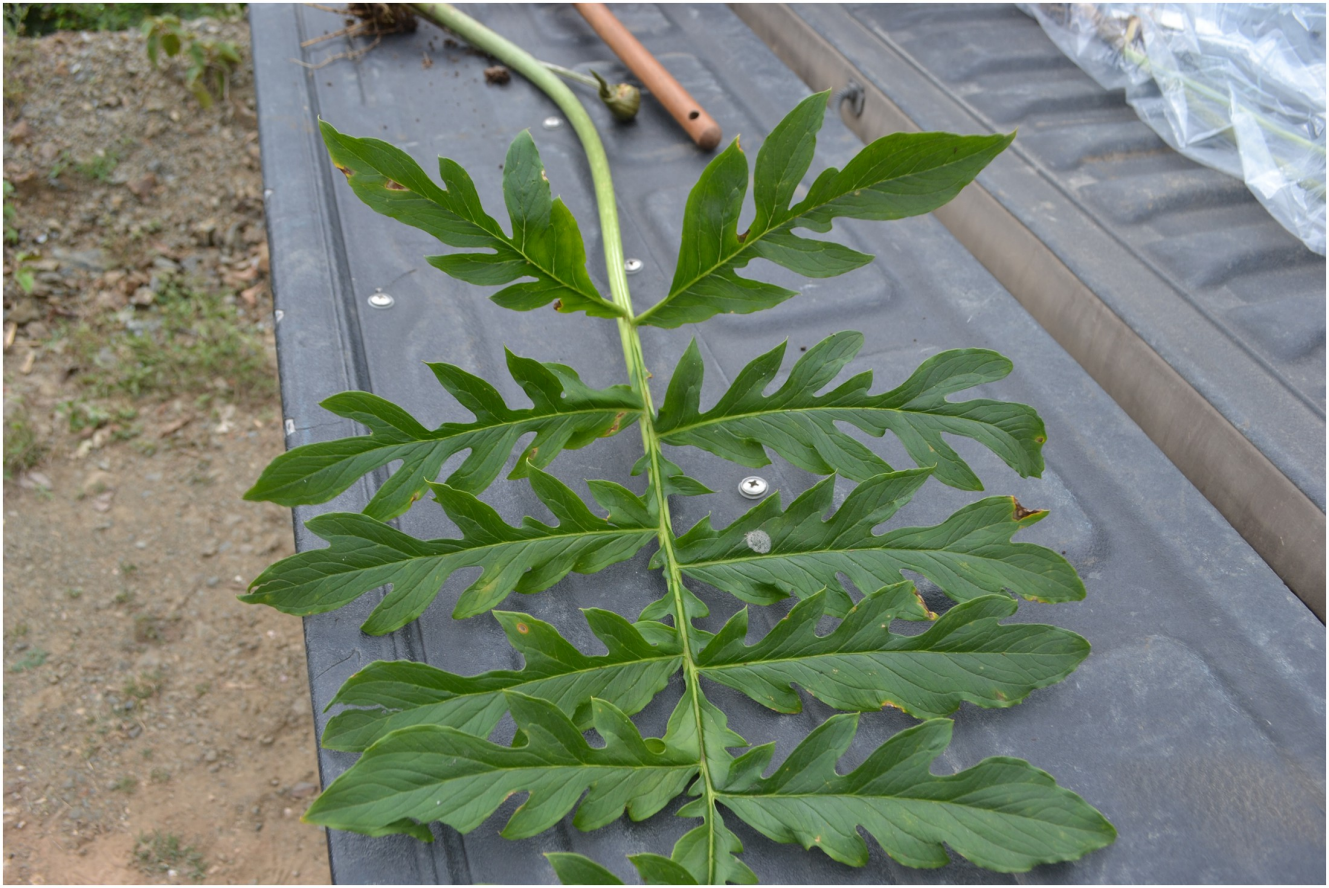
Leaf shape and leaf base of *Vivaria calvasensis*.

*Vivaria* is proposed as a new genus of Araceae (Aroidea, Spathicarpeae), and molecularly it is most closely related to two genera—*Gorgonidium* and *Incarum*—with which it forms a single clade. Below we detail the similar morphological characters which are common for the species in the clade, as well as the traits that distinguish *Vivaria* from its related species:

1. second order leaf incision is pinnatipartite, similar to *Gorgonidium* striatum and *Gorgonidium vermidicum*;
2. peduncle in *G*. *intermedium*, *G*. *striatum* and *G*. *vermidicum* is equal to or shorter than twice the spathe length, while in *I*. *pavonii* and *V*. *calvasensis* it is longer than twice the spathe length;
3. the spadix is completely adnate to the spathe in *G*. *intermedium* and *Vivaria*, up to 2/3 of the female portion adnate to the spathe, and the male portion at least partially free in *G*. *vermidicum* and *Incarum pavonii*, while in *G*. *striatum* the spadix is completely free;
4. spathe in *V*. *calvasensis* is convolute at base, forming a tube, while in *Gorgonidium* and *Incarum* it is cymbiform;
5. staminodes in *Vivaria calvasensis* are fused, while in *Gorgonidium* and *Incarum* they are free;
6. apical synandrodes are present in *Vivaria calvasensis*, while in *Gorgonidium* and *Incarum* they are absent;
7. synandrium in *Vivaria* is sessile, while in *Gorgonidium* and *Incarum* it is clearly pedicelled
8. the connective is inconspicuous and xattened in *Vivaria calvasensis*, while in *G*. *intermedium* and *G*. *striatum* it is conspicuously lobed to branched, and in *G*. *vermidicum* and *Incarum pavonii* it is rounded;
9. thecae shape in *Vivaria calvasensis* is oblong, while in *Gorgonidium* and *Incarum* it is rounded;
10. in *Vivaria calvasensis* the fruit color is white or pale green, while in *Gorgonidium* and *Incarum* it is purple or blackish-purple;
11. the base of the leaves in *Vivaria* is pinnatipartite, and the leaves of the seedlings are pinnatipartite (Fig 14), while in *Incarum* and *Gorgonidium* they are simple to cordate.

#### Conservation status

The new species has been encountered mainly in areas affected by extensive crop cultivation and livestock grazing (cattle and goats), as well as in moderately conserved areas. Being a seasonal species (the aerial part of the plant grows and shows itself in the rainy season), it is not well known by the local population, so it has no known uses or a vernacular name.

The currently known distribution range of *V*. *calvasensis* is small (less than 20 km^2^). An additional population might be present in a neighboring canton (Macará), although it has not been confirmed until now; even so, the distribution range would not increase substantially. The species has not been encountered in any national reserve or otherwise protected area, which would represent a risk for the conservation of the species. In the Loja province, we encountered no distributional records neither in the herbarium or GBIF database for species of the genera *Incarum* or *Gorgonidium*, which are closely related to *Vivaria calvasensis*. This suggests that the Spathicarpeae tribe of the Araceae family is rare in the study area. According to the criteria issued by the IUCN in 2012 [22], a taxon is considered vulnerable (Vu) and in danger of extinction when its populations face or show any of the following criteria: i) a marked decrease in the individuals of its populations, a criterion that we did not evaluate for *V*. *calvasensis* or ii) the estimated area of occupancy is less than 2000 km^2^ and the habitat is severely fragmented or it is known that it does not exist in more than 10 localities, this last criterion is the one we use to evaluate the taxon and place it within the vulnerable category (Vu).

## Discussion

The Spathicarpeae tribe (Araceae) was composed of 8 genera in 1997 [23]. Goncalves [19] reviewed the genus *Asterostigma*, specifically, *A*. *pavonii* and *A*. *integrifolium*, from which it derived two new monospecific genera, *Incarum* and *Croatiella*, respectively. This author [16] also reviewed the phylogeny of the whole tribe, including some species of the genus *Dieffenbachia*, and some species of the genus *Bognera*. Our results support the addition of a new genus, *Vivaria*, composed of one species.

Morphologically, *Vivaria* can be distinguished from *Incarum* by having pinnatipartite lamina, compared to the entire lamina that are characteristic to the latter. Both *Vivaria* and *Gorgonidium* have pinnate leaves, but the number of lobes differs between these two genera. In *Gorgonidium*, we can usually find between 5–7 lobes per pinna, while in *Vivaria* the number of lobes is greater 5 (7) -9. Structurally, the three genera have a solitary leaf and a floral peduncle, but in *Vivaria* the petiole reaches between 0.75–1 m, while in the two other genera, the petiole is smaller, being 0.21–0.45 m in Incarum [15], 0.06–0.1 m in *G*. *beckianun* and 0.36–0.4 m in *G*. *striatum*.

The description of *Vivaria calvasensis* enriches the so-called "Andean" clade of the Spathicarpeae tribe [21], in which *Gorgonidium*, *Incarum* and *Spathantheum* would be the most closely related genera to *Vivaria*.

The current distribution of the Spathicarpeae tribe includes areas of Ecuador climatically different from the current distribution of *Vivaria*. The other closely related species distributed in Ecuador, *I*. *pavonii*, is restricted to humid areas. Our findings expand not only the distribution range of Spathicarpeae in Latin America, but, more importantly, bring evidence supporting the replication of an adaptation pattern seen in other species of the clade which inhabit arid and semi-arid areas. In these species, individuals remain dormant most of the year, when humidity is low, and become active during the rainy season, when their leaves and reproductive organs grow.

The occurrence of *V*. would coincide with the northern limit of the so-called Pleistocene dry arc, i.e. a large area in the South American continent, considered as the geographical distribution zone of certain species typical for forests and dry scrub distributed in the inter Andean dry valleys of Ecuador and Peru [24].

Finally, Cusimano et al. [17] consider that the evolution of Araceae is a complex process, that has resulted in diverse forms of life, ranging from the aquatic *Pistia*, *Jasarum*, the strictly rheophytic schistoglottids, to land-based geophytes (*Vivaria calvasensis*) and epiphytes of various kinds.

## Material and methods

### Ethics statement

All the botanical collections included in this publication, were carried out under the project "FLORISTIC PROSPECTING AND STUDY OF CRYPTIC SPECIES IN THE DRY SCRUB OF SOUTHERN ECUADOR", carried out between 2012–2015, which was financed entirely by the Universidad Tecnica Particular of Loja (UTPL). AP was supported by the PROMETEO program of the National Secretariat of Science and Technology (SENECYT for its acronym in Spanish) public organization funded by the Ecuadorian Government.

None of the sampled areas were located inside protected areas, and no specific permit was required for these locations. The field studies did not involve endangered or protected species.

### Specimen collection and study site

**Fig 15:**
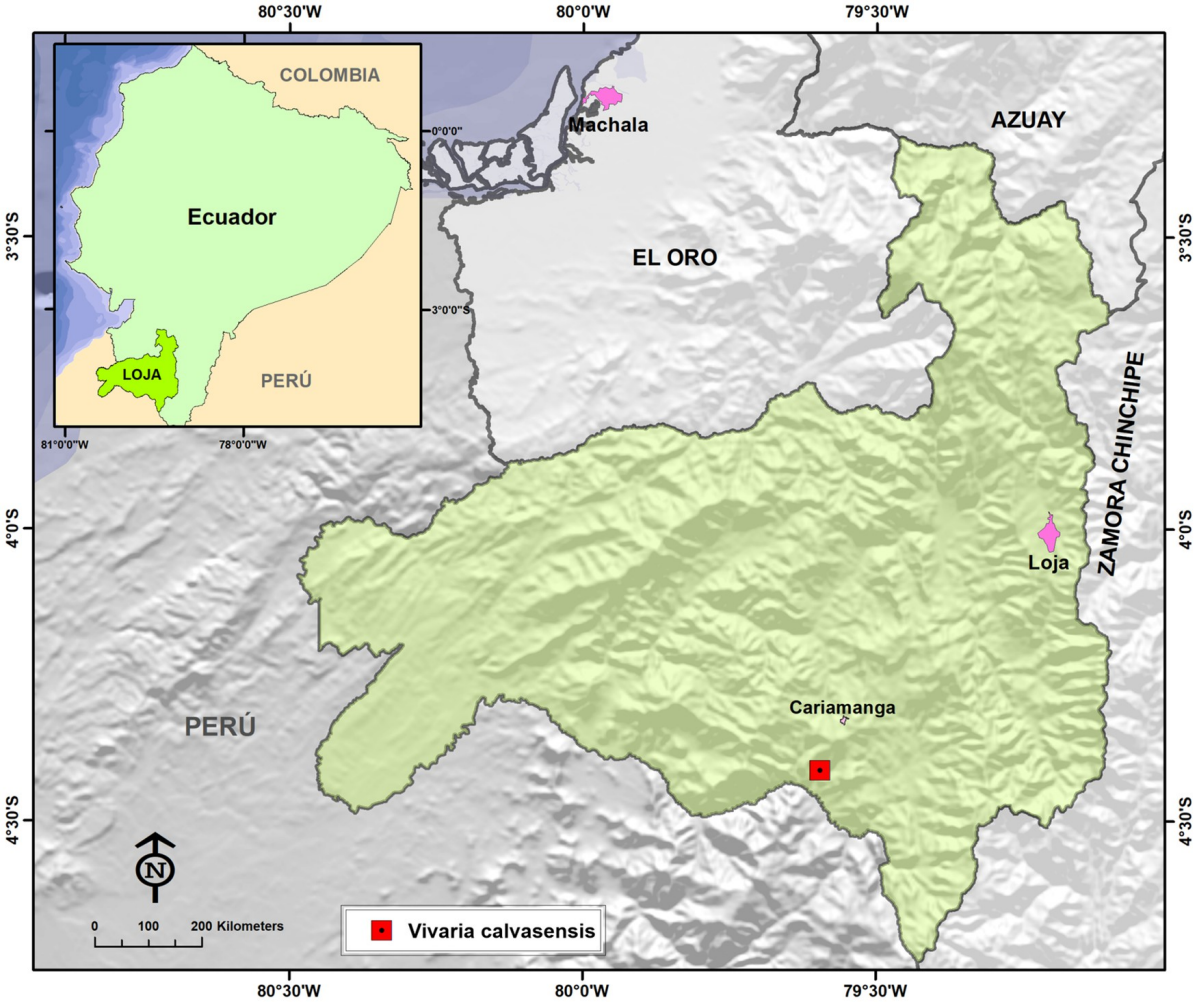
Location of the study area and geographic position of botanic collection of *Vivaria calvasensis*.

Field work was carried out between May 2012 and May 2015 visiting the dry inter-Andean valleys of southern Ecuador (Fig 15). During a botany trip financed by the HUTPL herbarium carried out in the Ecuadorian province of Loja, an infertile sample of a possible Araceae was collected, which was initially considered as belonging to the genus *Gorgonidium*, but with doubts about to the corresponding species. This led to a two-year search for fertile individuals of the species. The processed and labeled specimens (Type and paratypes) have been deposited in the Herbarium of the Universidad Tecnica Particular de Loja (HUTPL).

### Morphology

For the description of qualitative and quantitative morphological and floral characters we use some of those cited by Goncalvez et al. [16] in the study of the phylogeny of the Spathicarpae tribe (Table 3). We compare some of the characters of *Vivaria gen*. *et sp*. *nov*. with the same characters of the most closely-related species, in order to obtain a diagnosis that allows us to identify and differentiate the new genus from the rest of the genera in the tribe. Some important measurements were made directly on the collected specimens, and photographs were taken both in the field and in Herbarium.

**Table 3:** Characters used for the phenotypic analysis.

No.	Character	State
1	Habit	(0) Chameophyte; (1) geophytic cryptophyte; (2) helophytic cryptophyte; (3) hemiepiphyte
2	Growth pattern	(0) Evergreen; (1) seasonally deciduous
3	Stem	(0) Long, not tuberous and epigeous; (1) rhizomatous, not tuberous, epigeous or partially hypogeous; (2) strongly congest, tuberous, hypogeous
4	Architecture	(0) Monopodial leaves monomorphic; (1) monopodial leaves trimorphic
5	Leaves:	Number per individual (0) More than two per plant; (1) leaves solitary, rarelly two per plant
6	Venation	(0) Reticulated; (1) parallel
7	Trichomes in aerial parts	(0) Absent; (1) present
8	Basic leaf pattern	(0) Simple to cordate; (1) hastate to tripartite; (2) pedate **(3) pinnatipartite**
9	Second order leaf incisión	(0) Absent; (1) pinnatiWd; (2) bipinnatiWd
10	Peduncle	(0) Equaling or shorter than twice the spathe length; (1) longer than twice the spathe length
11	Spadix	(0) Completely free; (1) up to 2/3 of female portion adnathe to the spathe, but the male portion at least partially free; (2) spadix completely adnathe to the spathe
12	Spathe	(0) Cymbiform; (1) convolute at base, forming a tube; (2) expanded to retrovolute
13	Spathe persistence on fruiting	(0) Persistent up to the ripening of berries; (1) deciduous soon after anthesis
14	Staminodes in female flowers	(0) Absent; (1) present
15	Staminodes	(0) Free; (1) fused
16	Staminode shape	(0) Claviform; (1) spathuliform; (2) mushroom-shaped; (3) Wliform
17	Staminode ápex	(0) Smooth; (1) papillose
18	Intercalar synandrodes	(0) Absent; (1) present
19	Apical synandrodes	(0) Absent; (1) present (2) apical
20	Synandrium type	(0) Solid; (1) hollow
21	Synandrium	(0) Sessile to subsessile; (1) clearly pediceled.
22	Connective	(0) Inconspicuous and Xattened; (1) rounded; (2) conspicuous lobed to branched; (3) cylindric
23	Shape of thecae	(0) Oblong; (1) rounded
24	Insertion of thecae	(0) Embedded on connective; (1) sessile; (2) pedunculate
25	Positioning of thecae	(0) Parallel; (1) coherent
26	Alignment of thecae	(0) In line; (1) unordered
27	Locule number	(0) 1; (1) 2; (2) 3–5; (3) 6-many
28	Maximum number of ovules per locule	(0) 1; (1) 2; (2) 3-many
29	Fruit type	(0) Subcoriaceous pericarp; (1) membranous pericarp; (2) juicy pericarp
30	Fruit color	(0) Red or orange; (1) pale green, white or pale green; (2) purple or blackish-purple
31	Seed testa	(0) Smooth; (1) Rough
32	Endosperm	(0) Absent; (1) present
33	Posterior lobe	(0) Entire; (1) lobate or pinnate
34	Seedling leaf	(0) Cordate; (1) sagittate to hastate **(2) pinnatipartite**
35	Anterior lobe	(0) Entire; (1) lobate or pinnate

### Molecular analysis

DNA was extracted from two samples of fresh leaf segments using a Plant Mini Kit (Qiagen, Hilden, Germany), according to the manufacturer’s instructions. We amplified two genes, i.e., the plastid gene *matK* and the *trnL* intron with the *trnL-F* intergenic spacer (called simply *trnL-F*). The gene *matK* was amplified using the primer combination 371-F [25] and Trnk-2R [26]. The gene *trnL* was amplified using the primer combination Trnlf-C and Trnlf-F [27]. PCR conditions were as follows: initial denaturizing at 98 °C for 30 s; 30 cycles, each cycle consisting of one step of denaturizing at 98 °C for 10 s; annealing, at 60 ºC for 20 s and extension at 72 °C for 30 s; and final extension at 72 °C for 10 min. A control including PCR mix without DNA template was included in each PCR. Success of the PCR amplifications were tested in 0.7% agarose stained with GelRed. PCR products were purified using the QIAquick protocol (Qiagen). PCR products were sequenced bi-directionally by ABI 3730xl in Macrogen using the same primers as for PCR amplification.

### Phylogenetic analysis

Sequences were edited and consensus were generated using CodonCode Aligner Ver.5.0.2. BLAST [28] was used against the NCBI nucleotide database (GenBank; http://www.ncbi.nlm.nih.gov/) to check their similarity with published sequences. Phylogenetic analyses were performed according to Gonçalves et al. [16]. For that, the 35 sequences of each gene (*matK* and *trnL-F*) belonging to tribe Spathicarpeae studied by Gonçalves et al. [16] were downloaded from GenBank. Matrices containing sequences belonging to each gene were aligned separately using Mafft Ver. 6.620b [29] under the G-INS-i option. After that, we concatenated both alignments in a new matrix using MEGA11 software [30]. Then we performed a Bayesian approach based on Markov chain Monte Carlo (B/MCMC) and a maximum parsimony (MP) analysis. The B/MCMC analyses were conducted using the MrBayes Ver. 3.1 program [31]. We used the most complex substitution model available (GTR+I+G), including two runs each involving four incrementally heated Markov chains over 4 000 000 generations and using random starting trees [32–34]. Trees were sampled every 100 generations resulting in 40 000 trees from which the last 24 000 were used to compute a 50% majority-rule consensus tree, enabling the use of Bayesian Posterior Probabilities (BPP) as node support. Stationarity of the process and effective sample size (ESS) values were checked visually with the software Tracer 1.5 [35].

MP analysis for each alignment was calculated on MEGA11 software [36]. Heuristic searches with 1000 random taxon addition replicates were conducted with the tree-bisection-reconnection (TBR) method with search level 1 [37]. Clade support was inferred from bootstrapping [35] performed based on 1000 pseudoreplicates with the same settings as for the heuristic search. Only clades that received bootstrap support of greater than or equal to 70% in the MP or posterior probabilities of greater than or equal to 0.90 in the MrBayes analysis were considered to be well supported [37]. Phylogenetic trees were drawn using FigTree Ver. 1.4.3. (http://tree.bio.ed.ac.uk/software/figtree/).

### Nomenclature

The electronic version of this article in Portable Document Format (PDF) in a work with an ISSN or ISBN will represent a published work according to the International Code of Nomenclature for algae, fungi, and plants, and hence the new names contained in the electronic publication of a PLOS ONE article are effectively published under that Code from the electronic edition alone, so there is no longer any need to provide printed copies.

In addition, new names contained in this work have been submitted to IPNI, from where they will be made available to the Global Names Index. The IPNI LSIDs can be resolved and the associated information viewed through any standard web browser by appending the LSID contained in this publication to the prefix http://ipni.org/. The online version of this work is archived and available from the following digital repositories: PubMed Central, LOCKSS, RiUTPL, Redalyc, DOAJ.
